# Carbon nanodots revised: the thermal citric acid/urea reaction†

**DOI:** 10.1039/d0sc01605e

**Published:** 2020-07-17

**Authors:** Volker Strauss, Huize Wang, Simon Delacroix, Marc Ledendecker, Pablo Wessig

**Affiliations:** Department of Colloid Chemistry, Max-Planck-Institute of Colloids and Interfaces Am Mühlenberg 1 14476 Potsdam Germany volker.strauss@mpikg.mpg.de; Department of Technical Chemistry, Technical University Darmstadt Alarich-Weiss-Straße 8 64287 Darmstadt Germany; Institute of Chemistry, University of Potsdam Karl-Liebknecht-Str. 24-25 D-14476 Potsdam Germany

## Abstract

Luminescent compounds obtained from the thermal reaction of citric acid and urea have been studied and utilized in different applications in the past few years. The identified reaction products range from carbon nitrides over graphitic carbon to distinct molecular fluorophores. On the other hand, the solid, non-fluorescent reaction product produced at higher temperatures has been found to be a valuable precursor for the CO_2_-laser-assisted carbonization reaction in carbon laser-patterning. This work addresses the question of structural identification of both, the fluorescent and non-fluorescent reaction products obtained in the thermal reaction of citric acid and urea. The reaction products produced during autoclave–microwave reactions in the melt were thoroughly investigated as a function of the reaction temperature and the reaction products were subsequently separated by a series of solvent extractions and column chromatography. The evolution of a green molecular fluorophore, namely HPPT, was confirmed and a full characterization study on its structure and photophysical properties was conducted. The additional blue fluorescence is attributed to oligomeric ureas, which was confirmed by complementary optical and structural characterization. These two components form strong hydrogen-bond networks which eventually react to form solid, semi-crystalline particles with a size of ∼7 nm and an elemental composition of 46% C, 22% N, and 29% O. The structural features and properties of all three main components were investigated in a comprehensive characterization study.

## Abstract 2

Products of the thermal reaction of citric acid and urea have been identified as a complex mixture of fluorophores and particles.

## Graphical abstract

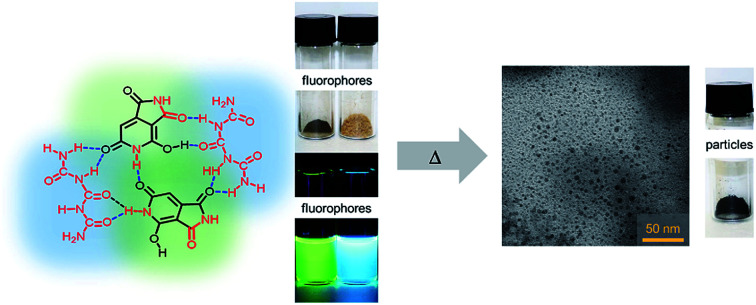

## Introduction

Small organic molecules are common starting materials for hydrothermal or pyrolysis reactions, bringing forth new compounds and materials with intriguing properties which are valuable for a wide range of applications.^1–7^ In particular, the so-called “carbon nanodots (CNDs)” have emerged as an interesting new class of photo- and electroactive materials and have been studied as components in functional systems and devices such as fluorescent sensors,^8,9^ light emitting diodes,^10^ drug delivery systems,^11,12^ charge storage systems,^13^ photosensitizers^9,14^*etc.* However, there has been a consensus among experts in the field that their structural identification and the correlation between their structure and optical properties are the most critical open questions that need to be answered.^15–17^

As an initial standard model for luminescent organic particles, a carbonized core bearing a number of functional groups, such as amides, carboxylates, hydroxyls and amines, was considered.^18,19^ The presence of a large number of functional groups would facilitate water solubility as shown in molecular dynamics simulations, which is usually not observed for graphitic nanoparticles.^20^ In the past few years, a variety of thermal reaction products from small organic precursors in the context of CNDs have been discussed and the identified or proposed product palette is wide ranged. For example, the fluorescence of the thermal CA/ethylene diamine reaction product was assigned to a specific molecular species.^21^ Other observations revealed the presence of pure graphitic C_3_N_4_ phases, when reacting a 1 : 6 mixture of sodium citrate/urea in an autoclave.^22^ These were documented by characteristic XRD peaks at 27.4 and 13.1° 2*θ*. In a later report, crystalline phases of β-C_3_N_4_ were identified by high resolution transmission electron microscopy studies.^23^ Recent literature demonstrates the existence of molecular fluorophores occurring in the product mixture.^24–28^ A recently published review article collects studies on revelations of molecular fluorophores as active species in CND samples based on typical reaction partners such as citric acid (CA)/ammonia, CA/urea (U), CA/cysteine, CA/ethylene diamine, CA/ethanolamine and other non-CA starting materials.^29^

The thermal reaction of CA and urea is of special importance in this regard, as it brings forth a variety of products with different structural and optical properties.^22,23,27,30,31^ For example, the reaction of CA and U involves the formation of citrazinic acid, a reaction that was described more than hundred years ago^32^ and, interestingly, it has been shown that citrazinic acid exhibits similar electronic deactivation behavior to that of the hydrothermal CA/urea-reaction product.^33^ Depending on the reaction conditions, however, other or additional products have been identified, such as crystalline particles and HPPT, a green fluorophore.^25,27,34^

Several purification or separation techniques have been proposed, including centrifugation, precipitation, extraction, chromatography and dialysis. Dialysis is the most widely used technique.^26,35–37^ Therefore, it has been demonstrated that the overall fluorescence in these samples originates from molecular fluorophores. The isolation of fluorophores is of pronounced importance to provide easy access to inexpensive and water-soluble photoactive compounds. In fact, new dyes and fluorophores, in particular those featuring a large Stokes shift, are essential for a range of applications, such as fluorescence lifetime imaging microscopy (FLIM), stimulated emission depletion microscopy (STED) and phase-fluorometric sensing (PFS).^38–40^

Besides water-soluble fluorophores, insoluble, non-fluorescent reaction products are also often produced during solvothermal or thermal reactions, which are typically discarded as carbonaceous particles or larger particles without further use.^36,41^ This insoluble fraction produced during the low-temperature thermal reaction of a CA/U mixture was found to be an excellent precursor material in our recently introduced laser-assisted carbonization process.^42^ A careful analysis of these products is, therefore, meaningful as it reveals the formation of an intermediate product on the way to a fully carbonized material. In general, the structural identification of such intermediate reaction products during low-temperature thermal synthesis may help to achieve a comprehensive understanding of carbonization reactions. The thermal CA/U reaction may be used as an example reaction, as the reaction conditions determine the reaction pathways to a broad product palette and the properties of the final carbonization product are a direct result of the structure of the low-temperature thermal intermediates.

In this study, we analyzed the thermal reaction of citric acid and urea in the melt by varying the reaction temperature. First, the reaction products were analyzed by means of optical spectroscopy and nuclear magnetic resonance (NMR) spectroscopy. Under any reaction conditions, the product consists of three main components, two soluble fractions, referred to as “thermally accessed fluorophores (TAFs)”, and an insoluble fraction (nanoparticles). The three fractions were separated and purified by a series of extractions and column chromatography. The isolated compounds were structurally characterized by electron spray ionization mass spectrometry (ESI-MS), 2D-correlation nuclear magnetic resonance (2D-NMR) and Fourier-transform infrared (FT-IR) spectroscopies, and thermogravimetric analysis (TGA). The insoluble fraction was characterized by means of transmission electron microscopy (TEM), FT-IR, X-ray diffraction (XRD), X-ray photoelectron spectroscopy (XPS) and TGA. Based on these results, a reaction mechanism is proposed and possible side reactions are discussed.

## Results and discussion

### Reaction conditions

**Fig. 1:**
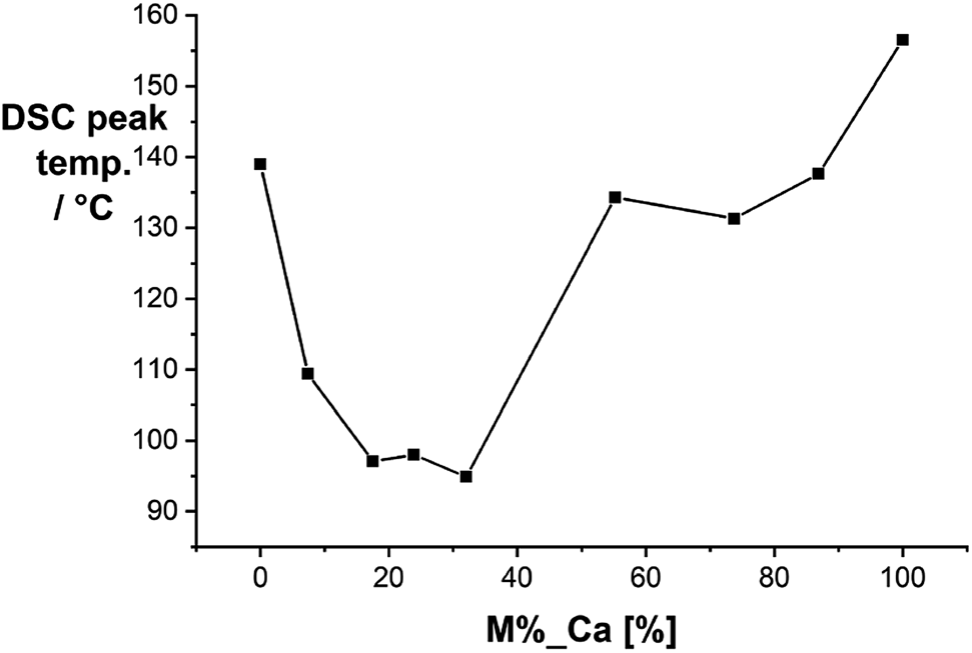
Melting points of citric acid/urea mixtures obtained by differential scanning calorimetry.

We started our investigation by analyzing the solid-state mixtures of citric acid and urea. Urea is a well-known hydrogen bond donor that forms strong eutectic mixtures with a variety of compounds.^43^ We performed differential scanning calorimetry (DSC) with mixtures of citric acid and urea at different compositions (Fig. 1). The mixtures form a eutectic system with melting points significantly lower than those of the pure compounds. The lowest melting points were measured for molar ratios between 1 : 5 and 1 : 3 (CA/U) at ∼95 °C.

Mixtures with a molar ratio of 1 : 3 were reacted at different temperatures ranging from 100 °C to 290 °C in a laboratory microwave. Besides the temperature, the reaction time was also found to have an influence on the reaction yields. For comparison, we performed every synthesis for 20 min. To ensure homogeneous blending of the precursors, the mixture was stirred at 100 °C for 5 min, and then the reaction was ramped up to the reaction temperature and kept for 20 min. At temperatures >130°, a yellow product with blue fluorescence was observed (Fig. S1†). However, significant amounts of unreacted precursors were still present in the products up to reaction temperatures of 150 °C, as shown in NMR experiments. At temperatures >150 °C, the visible fluorescence turns green. At 180 °C, the product yield increases dramatically and no reaction educts are contained in the product (Fig. S2†). In aqueous solutions of the raw products, a notable Tyndall scattering effect starts to occur at temperatures >190 °C.

**Fig. 2:**
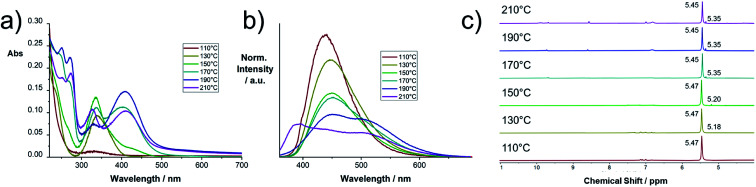
Characterization of selected raw products of the thermal CA/U reaction (0.013 mg mL^−1^) at 110–210 °C: (a) UV-vis absorption spectra in H_2_O; (b) fluorescence spectra in H_2_O obtained upon excitation at 350 nm; (c) ^1^H-NMR spectra recorded in DMSO-d6.

In Fig. 2a and b the electronic absorption and fluorescence spectra of selected raw products are shown. The spectra of all raw product samples synthesized at different temperatures between 100 and 290 °C are collected in Fig. S3.† In the low temperature regime between 100 and 140 °C, a distinct absorption peak at 337 nm evolves, which corresponds to the fluorescence peak at 440 nm. At 150 °C a second absorption peak with a maximum at 406 nm starts to evolve together with a set of peaks at <300 nm. The peak at 406 nm causes a second fluorescence peak at 523 nm. At temperatures >180 °C, a notable increase of the absorption at 406 nm with respect to the absorption at 337 nm occurs. Simultaneously, the absorption maxima shift from 337 and 406 to 327 and 411 nm, respectively. These changes occur along with the appearance of a new emission band with a maximum at 394 nm. This effect becomes more pronounced at temperatures >250 °C (Fig. S3†). Given the differences in the electronic absorption of the reaction products after the thermal CA/U reaction, we hypothesize a structural transition of the reaction product, the evolution of a new product, or electronic interaction between the species present. For example, a sample reacted at 230 °C contains all three products, since both the absorption peaks (327 and 411 nm) and all three emission peaks (394, 440, and 523 nm) are present. The correlation between the absorption and emission features has been previously assigned to different nitrogen doping levels of carbon nanodots or clusters of citrazinic acid.^19,28^

The ^1^H-NMR and ^13^C-NMR spectra of a selection of raw products synthesized at temperatures between 110 and 210 °C are shown in Fig. 2c and S2.† At low temperatures, a peak at 5.47 ppm arises, which remains dominant for any sample. The advent of this peak correlates with the evolution of the previously discussed absorption peak at 340 nm. At a reaction temperature of >150 °C, a peak at 5.18 is observed, which indicates the formation of the compound causing the absorption peak at 410 nm. At higher temperatures, the two peaks shift to 5.45 and 5.35, respectively. In the higher temperature samples (>150 °C), the educts, citric acid and urea, are entirely consumed, as no related NMR signals were observed (see Fig. S4†). Moreover, no evidence for the presence of citrazinic acid was found (see Fig. S5†).

### Separation

**Fig. 3:**
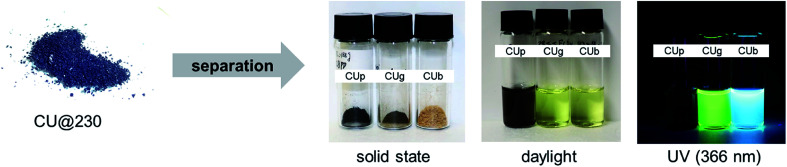
Separated products, **CUb**, **CUg**, and **CUp**, from the crude thermal reaction product of citric acid and urea; right: isolated reaction products in the solid state, in H_2_O in daylight and in H_2_O under UV light.

The product mixtures were separated by a series of solvent extractions followed by column chromatography (see Fig. S6† and the Experimental section). For example, a reaction of a 1 : 3 mixture of CA/U conducted at 230 °C yields a reaction product containing a minimum of three components. The three main components were isolated and characterized. Initially, the dry reaction product was dissolved in H_2_O_millipore_ and stirred well to dissolve the soluble part. The mixture was then centrifuged to separate the soluble part from the insoluble part. The supernatant was dried under reduced pressure and then re-dissolved in methanol (MeOH). The mixture was again centrifuged to remove insoluble materials and the obtained yellowish-brown supernatant was dried. The second washing step in MeOH helps to remove small amounts of residual insoluble particles and to obtain a pure fluorophore fraction. The product was then separated by column chromatography using silica gel as a stationary phase and H_2_O_millipore_ as the eluent yielding two fractions, a yellowish-brown fraction showing green emission (citric acid/urea-green: **CUg**) and a colorless fraction showing blue emission (citric acid/urea-blue: **CUb**) under UV light (Fig. 3). Other eluent/stationary phase combinations, such as acetonitrile or methanol failed to separate the reaction products. The precipitate from the initial centrifugation step was washed with H_2_O_millipore_ at 95 °C for 24 h and, then, again centrifuged. The larger fraction of the precipitate is a fine black powder (citric acid/urea-particles: **CUp**) that is dispersible in a range of solvents, even non-polar, such as CH_2_Cl_2_. The respective yields of the three main components depend on the reaction conditions. For example, a reaction at 180 °C with 20 min reaction time yields 46% **CUb**, 26% **CUg**, and 4% **CUp**. When performing the same reaction at 230 °C, 23% **CUb**, 35% **CUg**, and 36% **CUp** were obtained. The remaining mass corresponds to reaction side products or material losses (product mixtures) from the separation process.

The two fluorescent molecular fractions, **CUg** and **CUb**, are soluble in polar solvents, such as H_2_O or MeOH, and have clear NMR patterns; therefore, we refer to those as “thermally accessed fluorophores” (TAFs). The third fraction, **CUp**, is an insoluble, non-fluorescent material which consists of nanoparticles with sizes on the order of a few nanometers and a certain degree of crystallinity as shown by XRD measurements. The TAFs were analyzed by optical absorption and photoluminescence spectroscopies and 2D-correlative ^1^H- and ^13^C- and ^15^N-NMR spectroscopy, mass spectrometry (MS) and elemental analysis (EA) and X-ray photoelectron spectroscopy (XPS). **CUp** was analyzed by a set of characterization methods including XRD, XPS, TEM, EA, FT-IR, TGA and solid-state NMR.

### Characterization

#### TAFs

**Fig. 4:**
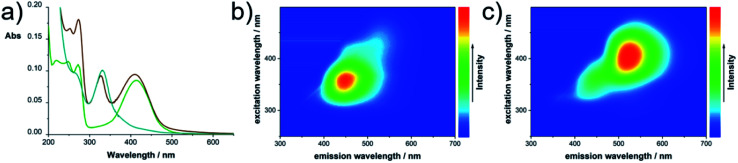
Optical characterization of TAFs: (a) absorbance of **CUg** (green), **CUb** (blue) and the unseparated raw product (brown) in H_2_O; (b) 2D photoluminescence plots of **CUb** and (c) **CUg** in H_2_O at room temperature.

The electronic absorption and emission spectra of the separated products show, indeed, that the peak at 330 nm originates from **CUb** (Fig. 4a). The corresponding fluorescence peak appears at 443 nm. **CUg** shows a set of absorption maxima at a wavelength of < 300 nm and a broad maximum at 410 nm (Fig. S7†) with a corresponding fluorescence maximum at 514 nm. Both fluorescence signals appear as single modes and originate from a single excitation peak as shown in the 2D photoluminescence plots in Fig. 4b and c. Notably, the fluorescence peak at 390 nm as observed in the raw products was not observed in the isolated fractions; therefore, we assume that it originated from the electronic interaction within the mixture or from a minor fraction that was lost during the separation. Fluorescence lifetimes were determined by time-correlated single-photon counting (TCSPC) (Fig. S8†). The fluorescence of **CUb** decays with two lifetime components of 4.1 and 9.5 ns in H_2_O and 3.1 and 7.6 ns in MeOH (Table S1†). The two lifetime components may derive from different species in the mixture of compounds (*vide infra*). The longer lifetimes of **CUb** in the more polar H_2_O are likely due to the higher polarity of their excited states. The opposite trend is observed for **CUg**, where single-exponential decays with lifetimes of 5.3 or 9.0 ns in H_2_O or MeOH, respectively, were obtained. The fluorescence quantum yields of **CUg** were determined by the gradient method to be 27% in H_2_O and 70% MeOH using Na-fluorescein as a standard (Fig. S9†). The results are summarized in Table S2.† Notably, **CUg** features remarkable photostability in solution. After 16 h illumination at 410 nm in H_2_O, the fluorescence intensity was still 100% (Fig. S10†). For **CUb**, a quantification of the optical parameters was difficult due to an intrinsic instability of the sample as shown in Fig. S10.†

First structural information was obtained by elemental analysis of the isolated fractions yielding 38% C, 13% N, 3% H and 44% O for **CUg**. This is in sound agreement with the predicted elemental composition of HPPT·2H_2_O (4-hydroxy-1*H*-pyrrolo[3,4-*c*]pyridine-1,3,6(2*H*,5*H*)-trione) as reported in the literature.^25^ For **CUb** an elemental composition of 25% C, 38% N, 6% H and 31% O was determined, which coincides very well with the predicted elemental composition of triuret or tetrauret: 25% C, 38% (37%) N, 4% H, and 33% (34%) O.

Mass spectrometric analysis shows a main molecular fraction with a mass of *m*/*z* = 179 for **CUg**, the expected mass for HPPT (Fig. S11†).^25^**CUb** was analyzed in both the positive ion mode and the negative ion mode. When looking at the significant noise in the ESI-MS pattern, **CUb** seems to contain a variety of small compounds (Fig. S12†). In the negative ion mode, a peak at *m*/*z* = 171 is dominant, which matches with a cyclic tetrauret species and the signal at *m*/*z* = 121 matches with the biuret–ammonium adduct. The dominant peak of **CUb** in the positive ion mode appears at *m*/*z* = 121 and minor fragments with signals of *m*/*z* = 149, 186, and 279 are detected. Important are the signals at *m*/*z* = 149 and 186 as these may originate from melamine·Na^+^ or melaminylguanidine·NH^4+^ (Fig. S13†). A minor peak at 128 indicates the presence of cyanuric acid.

**Fig. 5:**
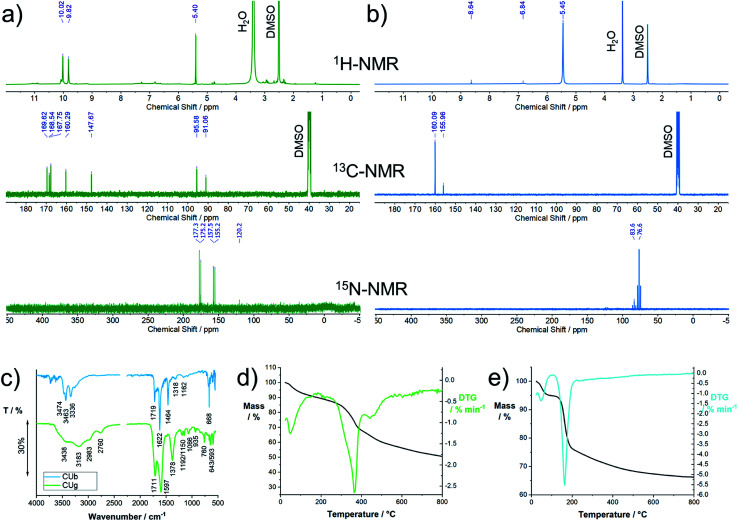
Structural characterization of TAFs, **CUg** and **CUb**, isolated from the reaction product of the thermal reaction of citric acid and urea: (a) ^1^H-, ^13^C-, and ^15^N-NMR spectra of **CUb** recorded in DMSO-d6; for the ^15^N-NMR spectrum, samples were enriched with ^15^N; (b) ^1^H-, ^13^C-, and ^15^N-NMR spectra of **CUg** recorded in DMSO-d6; for the ^15^N-NMR spectrum, samples were enriched with ^15^N; (c) solid state FT-IR spectra of **CUb** and **CUg**; (d) thermogravimetric analysis of **CUg**; (e) thermogravimetric analysis of **CUb**.

Characterization by nuclear magnetic resonance spectroscopy (NMR) was performed in DMSO-d6 or D_2_O. DMSO was found to be a good choice as a solvent for the characterization because proton exchange is suppressed to some degree. The ^1^H-NMR spectrum of **CUg** shows one dominant signal at 5.40 ppm and two peaks at 9.82 and 10.0 ppm (Fig. 5a). The ^13^C-NMR spectrum shows seven peaks at 169.6, 168.5, 167.75, 160.3, 147.7, 95.6, and 91.0 ppm. In the ^15^N-NMR spectrum, two peaks at 176.3 and 156.3 appear, indicating the presence of two imide-like nitrogens. The molecular structure was verified by ^13^C and ^15^N heteronuclear single quantum correlation (HSQC) and heteronuclear multiple-bond correlation (HMBC) spectroscopy experiments with ^15^N-enriched samples (Fig. S14†). Only one proton (5.40 ppm) is immediately bonded to carbon (95.6 ppm). Two protons (9.82 and 10.0 ppm) are directly bonded to nitrogens (176.3 and 156.3 ppm), respectively. Using heteronuclear multiple-bond correlation spectroscopy (HMBC), we identified the coupling of protons to ^13^C or ^15^N over three bonds within the structure (Fig. S15†) and were able to confirm the structure of HPPT.^25^

The correct tautomeric form of HPPT was confirmed by quantum mechanical calculations in a vacuum and polar solvents. The tautomer B with strong intramolecular H-bonding affinity was found to be the most stable (Fig. S16†).

In the ^1^H-NMR spectrum of **CUb**, a dominant peak at 5.45 and two weak peaks at 6.84 and 8.64 ppm are observed (Fig. 5b). The ^13^C-NMR spectrum shows two peaks at 160.1 and 156.0 ppm and the ^15^N spectrum shows two triplets centered at 76.6 and 83.6 ppm and a weak signal at 123 ppm. All of the ^15^N peaks appear in the amine-typical region. In neither of the ^1^H–^13^C 2D-correlation spectroscopies, HSQC and HMBC, coupling between protons and carbon was seen (Fig. S17 and S18†); therefore, carbon–hydrogen bonds are excluded. ^1^H–^15^N HSQC and HMBC experiments were performed with ^15^N-enriched samples to detect the coupling of ^1^H and ^15^N. A direct coupling between the proton signal at 5.45 and the amine-like ^15^N signal at 76.6 ppm is confirmed. Direct coupling was also observed between the ^1^H at 6.84 and the ^15^N at 83.6 ppm as well as the ^1^H at 8.64 ppm and the ^15^N at 123 ppm. Three-bond coupling between these ^1^H and ^15^N is also observed (Fig. S18†). In general, the NMR pattern resembles the expected pattern of oligomeric ureas such as triuret or tetrauret. A commercial reference for triuret is not available; the peak positions, however, match with a commercial biuret sample, for which similar shifts are expected (Fig. S19†). Based on these results, we assume small oligomeric ureas to be the dominant species in **CUb**. A positive “biuret test” confirmed the presence of oligomeric urea in **CUb** (Fig. S20†).

More structural information on **CUg** and **CUb** was obtained by FT-IR spectroscopy (Fig. 5c). For **CUg**, the broad peaks between 2700 and 3600 cm^−1^ indicate the presence of OH and NH stretching vibrations. The strong signals at 1711 and 1597 or 1719 and 1622 cm^−1^ are assigned to C=O and C=N stretching modes, respectively, and the peak at 1378 cm^−1^ is assigned to C–N stretching modes. Notably, the peaks appear significantly broadened in comparison to, for example, **CUb** or typical nucleobases or purine bases. Such a peak broadening is typically observed for molecules that undergo significant H-bonding. The FT-IR spectrum of **CUb** shows a set of distinct absorption bands at 3474, 3463, and 3336 cm^−1^, which are assigned to N–H stretching vibrations typically observed for primary amines (compared to urea, biuret, or melamine). The two sharp peaks at 1719 and 1622 cm^−1^ originate from C=O and C=N stretching modes, respectively. The peak at 1464 cm^−1^ derives from a C–N stretching vibration. In general, the spectrum resembles the FT-IR spectra of urea and its oligomers, as their dominant peaks occur in a similar fashion.

Thermogravimetric analysis provided information about the thermal stability of the isolated TAFs. **CUg** is stable up to a temperature of ∼300 °C with a peak decomposition temperature of 364 °C (Fig. 5d). In this temperature regime, the main decomposition products are NH^+^, CH_2_O_2_^+*^, CH_2_O^+*^, CHO_2_^+*^, CO_2_^+*^, CH_2_NH_2_^+^, CH^3^CN^+*^, and CH_3_CN^+*^ stemming from primary amines, CO_2_ and N-containing heterocycles (Fig. S21†). The results, presented in Fig. 5e, clearly show the low thermal stability of **CUb**. At a peak temperature of 164 °C, the sample decomposes. A simultaneous mass analysis of the decomposition products reveals that the major decomposition products stem from primary amines and low-molecular-weight carbon species. The evaporation products are NH^+^, NH_2_^+^, NH_3_^+*^, CH_2_O_2_^+*^, CHO_2_^+*^, CO_2_^+*^, and CH_2_NH_2_^+^ (Fig. S22†). Notably, the decomposition of **CUb** occurs at a significantly higher temperature of ∼210 °C in combination with **CUg**. A TGA profile of a raw mixture prior to column chromatography is shown in Fig. S23.†

The elemental composition and the structural features of the proposed products, **CUg** and **CUb**, were confirmed by X-ray photoelectron spectroscopy (Fig. S24†). In **CUg**, the expected binding situation as for HPPT is observed, with contributions from C–N, C=N, C=O, and C–NH. Adsorbed H_2_O causes a strong signal maximizing at 531 eV. The C–N or C=N features are the dominant X-ray absorption features in **CUb**. All peaks, the C_1s_, N_1s_ and O_1s_ peaks, in **CUb** show a predominance of C=N, C–N, C–O, and C=O, as present in oligomeric ureas.

#### Particles

The insoluble fraction of the reaction product, **CUp**, was analyzed by means of a set of characterization methods. **CUp** was obtained by washing the centrifugate with water at 95 °C several times. About 10 wt% of the material removed during the washing process is composed of molecular compounds such as HPPT (Fig. S25†). The remaining material appears as a fine black powder without apparent fluorescence in dispersion. In alkaline solutions or basic solvents, such as DMSO or DMF, **CUp** seemingly solubilizes with an apparent color change to brown. Upon re-dispersion of **CUp** into aqueous dispersion, slight yellow-brown coloring of the solvent is noted. This effect is even more pronounced in DMSO or dimethylformamide (DMF). A concentration dependent UV-vis and fluorescence analysis of **CUp** in H_2_O, shown in Fig. S26†, reveals strong auto-quenching effects in solution. Moreover, the UV-vis absorption pattern shows a strong plasmonic background absorption trailing from the UV into the NIR. Two broad maxima at ∼345 and 430 nm are observed in the UV-vis spectra (Fig. S26†). In principle, this absorption pattern resembles the one of **CUg**, however, with large red shifts of ∼15 and 25 nm. The fluorescence intensity is rather low and maximizes at 463 nm upon excitation at 350 nm. Concentration dependent spectroscopic assays reveal a strong tendency towards aggregation-induced quenching at concentrations above 4 mg L^−1^.

**Fig. 6:**
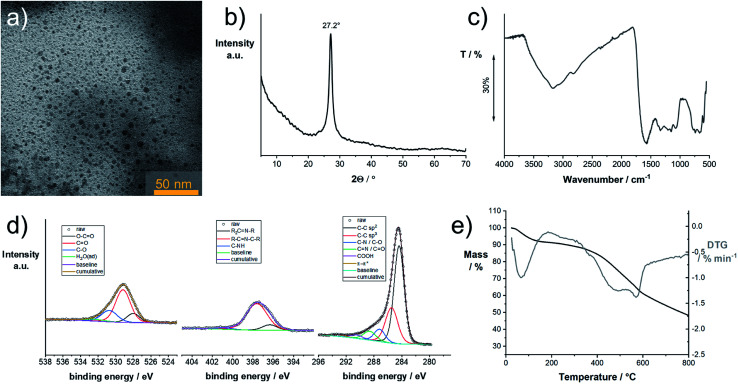
Characterization of the insoluble reaction product, **CUp**, isolated from the reaction product of the thermal reaction product of citric acid and urea: (a) transmission electron micrograph of **CUp** deposited from a dispersion in DMSO; (b) powder X-ray pattern of **CUp**; (c) Fourier-transform infrared spectrum of **CUp**; (d) X-ray photoelectron spectra of **CUp** with emphasis on the C_1s_, N_1s_, and O_1s_ regions; (e) thermogravimetric analysis of **CUp** in an inert atmosphere.

NMR experiments were performed in basic solution and DMSO but did not show any signals. Elemental analysis yields a composition of 46% C, 22% N, and 29% O. We examined the product by transmission electron microscopy (TEM). The TEM samples were prepared from diluted dispersions in DMSO. In Fig. 6a, a representative TEM image of **CUp** is shown. Throughout the investigated areas, particles with sizes of about 2–7 nm were observed. These are also seen in samples prepared from aqueous solutions, however, strongly aggregated. The X-ray powder diffraction pattern of **CUp** in Fig. 6b shows a peak at 27.2° 2*θ* (Cu K_α_) indicating the presence of a graphitic (002) lattice spacing typically seen for graphitic or graphitic carbon nitride samples. The particle sizes observed in TEM match with the mean size of the graphitic domains as determined by the peak width using the Scherrer equation.

FT-IR spectroscopy reveals the presence of OH groups within the material (Fig. 6c). Furthermore, a large contribution to the IR absorption is coming from C=O stretching vibrations. Other peaks indicate the presence of C–N and C=N groups. Similar to the observation made for **CUg**, the IR-absorption peaks appear rather broad, which indicate strong H-bonding within the sample.

A more comprehensive picture about the composition and the type of bonding in the material was provided by X-ray photoelectron spectroscopy (XPS). The fitted spectra with focus on the C_1s_, N_1s_, and O_1s_ region are shown in Fig. 6d. Fitting of the C_1s_ signal suggests an abundance of sp^2^ carbons.^44,45^ Other signals with lower intensity are assigned to C–C (sp^3^), C=N and C–O. The C=N signals are also observed in the N_1s_ region at 398 eV. A set of three signals in the O_1s_ region is assigned to C–O, C=O, and O–C=O bonds. The thermal stability was determined with TGA in an inert atmosphere (Fig. 6e). Decomposition starts at ∼324 °C and is accomplished at 610 °C.

### Proposed reaction mechanism

**Fig. 7:**
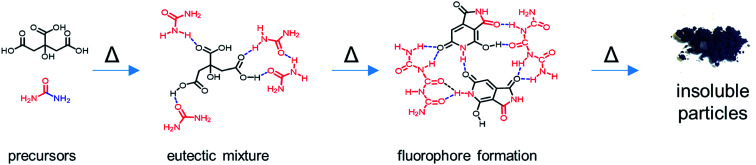
Proposed reaction mechanism of citric acid and urea to produce solid organic nanoparticles *via* molecular intermediates.

Taking the results from the structural characterization into consideration, we propose a potential reaction mechanism. Citric acid and urea form a solid eutectic mixture at room temperature (Fig. 7). H-Bonds between the two species are responsible for a drastic reduction of the melting point to ∼95 °C. The H-bond networks formed between citric acid and urea are expected to be rather complex due to the amphiphilic H-bond formation properties of both compounds. Upon heating to the reaction temperatures, urea dissociates into isocyanic acid and ammonia and condenses with citric acid to form citrazinic acid, which immediately reacts with isocyanic acid to form HPPT according to a mechanism provided in the literature.^25^ Notably, as shown in the literature, the citric acid/urea reaction in H_2_O yields different reaction products as the reaction from citrazinic acid to HPPT is suppressed by H_2_O.^25^ Simultaneously, excess urea condenses into oligomeric ureas, such as triuret or tetrauret, as well as small amounts of side products such as melamine or guanidine, the typical condensation products of urea.^46,47^ The unstable blue fluorescence likely originates from conjugated oligomeric ureas or isolated CN ring structures formed from urea which are stabilized by their oligomers. These reaction intermediates form a strong H-bond network. Upon further heating, both **CUb** and **CUg** are consumed in a further condensation reaction to form **CUp**, which appears as insoluble particles. H-Bonding between all three components facilitates the stable dispersion of particles. Strong H-bonding is still observed in **CUg** and interactions between **CUb** and **CUg** are inferred from TGA measurements and shifts in the NMR spectra. In synergy with **CUg**, the thermal decomposition of **CUb** occurs at 210 °C instead of 164 °C (Fig. S23†). Significant shifts in the ^1^H-NMR spectra were observed in the mixture of both compounds (Fig. S27†), which strongly suggests that the H-bond networks formed between **CUb** and **CUg** increase their thermal stability. Considering the relative reaction yields of the three main compounds in the reaction at 180 °C and 230 °C, we assume a consumption of both **CUg** and **CUb** on account of **CUp**. These observations coincide with the results of other reports on the same reaction.^28^

This assumption is supported by the analysis of the elemental compositions. With respect to the determined compositions of the three components, **CUp** has the highest carbon content and a medium nitrogen content. On the one hand, XPS and FT-IR measurements show that large amounts of C–N and C=N bonds are present. The XRD peak at 2*θ* of 27.2° and the fact that the dominant carbon species is sp^2^-hybridized, as shown in XPS experiments, point to the presence of nitrogen-containing graphitic domains in **CUp**.

Notably, in our experiments, we focused on CA/U reaction mixtures with a molar ratio of 1 : 3. It is feasible that higher amounts of urea lead to the predominant formation of C_3_N_4_ clusters in the reaction product.^23,34^ Isocyanic acid, the initial reactive species formed, is known to react with cyanuric acid and carbon nitrides.^48^ The covalent incorporation of **CUg** into such nitrogen-containing graphitic domains is feasible as absorption features resembling those of **CUg** are still observed in the solution phase of purified **CUp**.^27,49^ H-Bonding between excess TAFs and **CUp** may support the solubilization of the latter. In the crude mixture, molecular compounds such as **CUg** and **CUb** act as surfactants and prevent the aggregation of **CUp**.

Condensation products of only citric acid are largely suppressed by the excessive presence of urea. When heating only citric acid in neutral aqueous solution, typically a brown or orange product is obtained which is soluble in water. This brown product is most likely a polymer formed by the fusion of citric acid or aconitic acid molecules. Aconitic acid is formed at a temperature of ∼120 °C upon elimination of the hydroxyl group and a proton.^50^ Upon addition of a base, the carboxylic acid groups are deprotonated and upon further heating, a decarboxylation reaction occurs which leads to the reduction and aromatization of the citric acid polymer.^51,52^ The product based on pure citric acid exhibits a single absorption maximum at ∼360 nm and the fluorescence quantum yields are reported to be rather low.^51^

## Conclusions

The thermal reaction of citric acid and urea in the melt was thoroughly investigated. The composition of the reaction product is strongly dependent on the reaction parameters such as temperature, time, and composition. In this study, the reaction products were analyzed at a constant reaction time and different temperatures by means of absorption and fluorescence and NMR spectroscopies. Three main reaction products are formed during the reaction depending on the temperature, namely two molecular fluorophores and non-fluorescent carbonaceous particles. These products were isolated by a series of extractions and chromatography and thoroughly characterized in a comprehensive study. Based on the results, a potential reaction mechanism was proposed in which the nanoparticles are a product of the intermediately formed fluorophores.

The results presented herein demonstrate the complexity of the thermal citric acid/urea reaction. The raw reaction product should be seen as a complex mixture with symbiotic properties, similar to natural products such as litmus. Based on the results presented and recent literature, a clear distinction between thermally accessed fluorophores and carbonaceous nanoparticles should be made and thorough separation may help to improve their performance in various applications.

These insights not only help to understand the origin of the fluorescence in pyrolyzed organic materials, in general, but also provide easy access to a new class of highly inexpensive, water-soluble fluorescent molecules, such as HPPT, which is an interesting candidate for biological or fluorescence imaging and sensing applications due to its excellent photophysical properties. Moreover, as demonstrated in earlier studies, non-fluorescent organic nanoparticles are excellent precursors for our recently developed laser-assisted carbonization process. The structural information from this study helps to understand the fundamental chemical requirements for a good carbon precursor for this laser-assisted carbonization process.

## Experimental section

*Synthesis*: Two stock solutions of CA/U with molar ratios 1 : 3 and 1 : 5 were prepared with total concentrations of 100 or 84 g L^−1^, respectively. 4 mL of the stock solutions were added to the microwave vessel and H_2_O was evaporated under reduced pressure in a vacuum oven. The solid mixture was pre-stirred for 5 min at 90 °C to ensure complete melting. Then the microwave power was increased to reach the reaction temperature. The reaction temperature was held for 20 min. The product was collected and analyzed or further purified. *Melting point analysis:* Homogeneous mixtures of Ca and U were produced by solubilizing both components (*ca.* 1 g in total) in 1 mL H_2_O. H_2_O was then slowly evaporated under reduced pressure at room temperature. The resulting homogeneous mixtures were then analyzed by differential scanning calorimetry. *Chromatographic separation:* The separated fractions were collected and condensed. The solution was filtered to remove any solid silica that was washed from the column. Each fraction was dried under reduced pressure. *Transmission electron microscopy* was performed using an EM 912 Omega from Zeiss operating at 120 kV. To prepare the TEM samples, the carbon material was dispersed in methanol by sonication for 10 min and 5 μL droplets of the dispersion were placed on a carbon-coated copper TEM grid and dried at room temperature. *Thermogravimetric analysis* was performed using a Thermo Microbalance TG 209 F1 Libra (Netzsch, Selb, Germany). A platinum crucible was used for measuring 10 ± 1 mg of samples under a nitrogen flow of 20 mL min^−1^ and a purge flow of 20 mL min^−1^ at a heating rate of 10 K min^−1^. *Elemental analysis* was performed with a vario MICRO cube CHNOS elemental analyzer (Elementar Analysensysteme GmbH). The elements were detected with a thermal conductivity detector (TCD) for C, H, N and O and an infrared (IR) detector for sulfur. *UV-vis-NIR absorption* measurements were performed with a Specord 210 plus from Analytik Jena using 10 mm quartz cuvettes. *Fluorescence* measurements were performed with a Fluoromax 4 from Horiba. The fluorescence decays were measured using a Single Photon Counting Controller Fluorohub (Horiba Jobin Yvon) operating in the time-correlated single-photon-counting (TCSPC) mode. A nanosecond pulsed diode laser NanoLED-450 (Horiba Jobin Yvon) with a pulse width of 1.3 ns, *λ*_ex_ = 447 nm and a repetition rate of 1 MHz was used for excitation. *Fourier-transform infrared* measurements were performed using a Nicolet iS 5 FT-IR-spectrometer in conjunction with an iD5 ATR unit from Thermo Fisher Scientific. *X-ray diffraction* was performed on a Bruker D8 Advance diffractometer in the Bragg–Brentano mode at the Cu Kα wavelength. *Nuclear-magnetic resonance spectroscopy* was performed on a 400 MHz Bruker Ascend 400 or a 700 MHz Bruker Ascend 700. *X-ray photoelectron spectroscopy* was performed on a Quantera II (Physical Electronics, Chanhassen, MN, USA). A monochromatic Al Kα X-ray source (1486.6 eV) operating at 15 kV and 25 W was applied. Each measured spot was sputtered with Ar-ions (1 keV) for 30 seconds for surface cleaning. The C=C, sp^2^ carbon peak was referenced to 284.4 eV according to the literature.^53–56^ Casa XPS was used for fitting. *Geometries* of tautomers A–C were pre-optimized using the MMFF94x^57^ forcefield and the program MOE 2008.10.^58^ Based on these geometries, DFT calculations were performed using the program package Gaussian 16 (ref. 59) with the hybrid functional M062X^60^ and the basis set def2-TZVP.^61^ Dispersion correction was performed by using the empirical dispersion developed by Grimme.^62^ Moreover, calculations were performed in the presence of a solvent (MeOH, H_2_O) using the PCM model developed by Tomasi.^63^ For all obtained geometries, frequency analyses were performed to ensure that they are minima and to calculate the zero-point vibrational energy.

## Conflicts of interest

There are no conflicts to declare.

## Supplementary Material

SC-011-D0SC01605E-s001

## References

[cit1] Choi Y., Choi Y., Kwon O.-H., Kim B.-S. (2018). Carbon Dots: Bottom-Up Syntheses, Properties, and Light-Harvesting Applications. Chem.–Asian J..

[cit2] Das R., Bandyopadhyay R., Pramanik P. (2018). Carbon Quantum Dots from Natural Resource: A Review. Mater. Today Chem..

[cit3] Wu Z.-Y., Xu S.-L., Yan Q.-Q., Chen Z.-Q., Ding Y.-W., Li C., Liang H.-W., Yu S.-H. (2018). Transition Metal–Assisted Carbonization of Small Organic Molecules toward Functional Carbon Materials. Sci. Adv..

[cit4] Xia C., Zhu S., Feng T., Yang M., Yang B. (2019). Evolution and Synthesis of Carbon Dots: From Carbon Dots to Carbonized Polymer Dots. Adv. Sci..

[cit5] Titirici M.-M., Thomas A., Antonietti M. (2007). Back in the Black: Hydrothermal Carbonization of Plant Material as an Efficient Chemical Process to Treat the CO2 Problem?. New J. Chem..

[cit6] Antonietti M., Oschatz M. (2018). The Concept of “Noble, Heteroatom-Doped Carbons,” Their Directed Synthesis by Electronic Band Control of Carbonization, and Applications in Catalysis and Energy Materials. Adv. Mater..

[cit7] Demir-Cakan R., Baccile N., Antonietti M., Titirici M.-M. (2009). Carboxylate-Rich Carbonaceous Materials via One-Step Hydrothermal Carbonization of Glucose in the Presence of Acrylic Acid. Chem. Mater..

[cit8] Yoo D., Park Y., Cheon B., Park M.-H. (2019). Carbon Dots as an Effective Fluorescent Sensing Platform for Metal Ion Detection. Nanoscale Res. Lett..

[cit9] Margraf J. T., Lodermeyer F., Strauss V., Haines P., Walter J., Peukert W., Costa R. D., Clark T., Guldi D. M. (2016). Using Carbon Nanodots as Inexpensive and Environmentally Friendly Sensitizers in Mesoscopic Solar Cells. Nanoscale Horiz..

[cit10] Song S. H., Jang M.-H., Chung J., Jin S. H., Kim B. H., Hur S.-H., Yoo S., Cho Y.-H., Jeon S. (2014). Highly Efficient Light-Emitting Diode of Graphene Quantum Dots Fabricated from Graphite Intercalation Compounds. Adv. Opt. Mater..

[cit11] Hasenöhrl D. H., Saha A., Strauss V., Wibmer L., Klein S., Guldi D. M., Hirsch A. (2017). Bulbous Gold–Carbon Nanodot Hybrid Nanoclusters for Cancer Therapy. J. Mater. Chem. B.

[cit12] Tang J., Kong B., Wu H., Xu M., Wang Y., Wang Y., Zhao D., Zheng G. (2013). Carbon Nanodots Featuring Efficient FRET for Real-Time Monitoring of Drug Delivery and Two-Photon Imaging. Adv. Mater..

[cit13] Wang C., Strauss V., Kaner R. B. (2019). Carbon Nanodots for Capacitor Electrodes. Trends Chem..

[cit14] Martindale B. C. M., Hutton G. A. M., Caputo C. A., Prantl S., Godin R., Durrant J. R., Reisner E. (2017). Enhancing Light Absorption and Charge Transfer Efficiency in Carbon Dots through Graphitization and Core Nitrogen Doping. Angew. Chem., Int. Ed..

[cit15] Yao B., Huang H., Liu Y., Kang Z. (2019). Carbon Dots: A Small Conundrum. Trends Chem..

[cit16] Sciortino A., Cannizzo A., Messina F. (2018). Carbon Nanodots: A Review—From the Current Understanding of the Fundamental Photophysics to the Full Control of the Optical Response. C.

[cit17] Tian P., Tang L., Teng K. S., Lau S. P. (2018). Graphene Quantum Dots from Chemistry to Applications. Mater. Today Chem..

[cit18] Qu S., Wang X., Lu Q., Liu X., Wang L. (2012). A Biocompatible Fluorescent Ink Based on Water-Soluble Luminescent Carbon Nanodots. Angew. Chem., Int. Ed..

[cit19] Strauss V., Kahnt A., Zolnhofer E. M., Meyer K., Maid H., Placht C., Bauer W., Nacken T. J., Peukert W., Etschel S. H. (2016). *et al.*, Assigning Electronic States in Carbon Nanodots. Adv. Funct. Mater..

[cit20] Strauss V., Margraf J. T., Dolle C., Butz B., Nacken T. J., Walter J., Bauer W., Peukert W., Spiecker E., Clark T. (2014). *et al.*, Carbon Nanodots: Toward a Comprehensive Understanding of Their Photoluminescence. J. Am. Chem. Soc..

[cit21] Song Y., Zhu S., Zhang S., Fu Y., Wang L., Zhao X., Yang B. (2015). Investigation from Chemical Structure to Photoluminescent Mechanism: A Type of Carbon Dots from the Pyrolysis of Citric Acid and an Amine. J. Mater. Chem. C.

[cit22] Zhou J., Yang Y., Zhang C. Y. (2013). A Low-Temperature Solid-Phase Method to Synthesize Highly Fluorescent Carbon Nitride Dots with Tunable Emission. Chem. Commun..

[cit23] Messina F., Sciortino L., Popescu R., Venezia A. M., Sciortino A., Buscarino G., Agnello S., Schneider R., Gerthsen D., Cannas M. (2016). *et al.*, Fluorescent Nitrogen-Rich Carbon Nanodots with an Unexpected β-C 3 N 4 Nanocrystalline Structure. J. Mater. Chem. C.

[cit24] Hill S. A., Benito-Alifonso D., Davis S. A., Morgan D. J., Berry M., Galan M. C. (2018). Practical Three-Minute Synthesis of Acid-Coated Fluorescent Carbon Dots with Tuneable Core Structure. Sci. Rep..

[cit25] Kasprzyk W., Świergosz T., Bednarz S., Walas K., Bashmakova N. V., Bogdał D. (2018). Luminescence Phenomena of Carbon Dots Derived from Citric Acid and Urea – a Molecular Insight. Nanoscale.

[cit26] Hinterberger V., Damm C., Haines P., Guldi D. M., Peukert W. (2019). Purification and Structural Elucidation of Carbon Dots by Column Chromatography. Nanoscale.

[cit27] Schneider J., Reckmeier C. J., Xiong Y., von Seckendorff M., Susha A. S., Kasák P., Rogach A. L. (2017). Molecular Fluorescence in Citric Acid-Based Carbon Dots. J. Phys. Chem. C.

[cit28] Zholobak N. M., Popov A. L., Shcherbakov A. B., Popova N. R., Guzyk M. M., Antonovich V. P., Yegorova A. V., Scrypynets Y. V., Leonenko I. I., Baranchikov A. Y. (2016). *et al.*, Facile Fabrication of Luminescent Organic Dots by Thermolysis of Citric Acid in Urea Melt, and Their Use
for Cell Staining and Polyelectrolyte Microcapsule Labelling. Beilstein J. Nanotechnol..

[cit29] Qu D., Sun Z. (2020). The Formation Mechanism and Fluorophores of Carbon Dots Synthesized via a Bottom-up Route. Mater. Chem. Front..

[cit30] Qu S., Zhou D., Li D., Ji W., Jing P., Han D., Liu L., Zeng H., Shen D. (2016). Toward Efficient Orange Emissive Carbon Nanodots through Conjugated Sp 2 -Domain Controlling and Surface Charges Engineering. Adv. Mater..

[cit31] Sciortino L., Sciortino A., Popescu R., Schneider R., Gerthsen D., Agnello S., Cannas M., Messina F. (2018). Tailoring the Emission Color of Carbon Dots through Nitrogen-Induced Changes of Their Crystalline Structure. J. Phys. Chem. C.

[cit32] Sell W. J., Easterfield T. H. (1893). LXXIII.—Studies on Citrazinic Acid. Part I. J. Chem. Soc., Trans..

[cit33] Wang W., Wang B., Embrechts H., Damm C., Cadranel A., Strauss V., Distaso M., Hinterberger V., Guldi D. M., Peukert W. (2017). Shedding Light on the Effective Fluorophore Structure of High Fluorescence Quantum Yield Carbon Nanodots. RSC Adv..

[cit34] Sciortino A., Mauro N., Buscarino G., Sciortino L., Popescu R., Schneider R., Giammona G., Gerthsen D., Cannas M., Messina F. (2018). β-C 3 N 4 Nanocrystals: Carbon Dots with Extraordinary Morphological, Structural, and Optical Homogeneity. Chem. Mater..

[cit35] Zhou J., Yang Y., Zhang C. (2013). A Low-Temperature Solid-Phase Method to Synthesize Highly Fluorescent Carbon Nitride Dots with Tunable Emission. Chem. Commun..

[cit36] Nandy A., Kumar A., Dwivedi S., Pal S. K., Panda D. (2019). Connecting the Dots of Carbon Nanodots: Excitation (In)Dependency and White-Light Emission in One-Step. J. Phys. Chem. C.

[cit37] Wang Y., Zhu Y., Yu S., Jiang C. (2017). Fluorescent Carbon Dots: Rational Synthesis, Tunable Optical Properties and Analytical Applications. RSC Adv..

[cit38] Li Z., Askim J. R., Suslick K. S. (2019). The Optoelectronic Nose: Colorimetric and Fluorometric Sensor Arrays. Chem. Rev..

[cit39] Blom H., Widengren J. (2017). Stimulated Emission Depletion Microscopy. Chem. Rev..

[cit40] Borst J. W., Visser A. J. W. G. (2010). Fluorescence Lifetime Imaging Microscopy in Life Sciences. Meas. Sci. Technol..

[cit41] Miao X., Qu D., Yang D., Nie B., Zhao Y., Fan H., Sun Z. (2018). Synthesis of Carbon Dots with Multiple Color Emission by Controlled Graphitization and Surface Functionalization. Adv. Mater..

[cit42] Strauss V., Marsh K., Kowal M. D., El-Kady M. F., Kaner R. B. (2018). A Simple Route to Porous Graphene from Carbon Nanodots for Supercapacitor Applications. Adv. Mater..

[cit43] Smith E. L., Abbott A. P., Ryder K. S. (2014). Deep Eutectic Solvents (DESs) and Their Applications. Chem. Rev..

[cit44] Ganguly A., Sharma S., Papakonstantinou P., Hamilton J. (2011). Probing the Thermal Deoxygenation of Graphene Oxide Using High-Resolution In Situ X-Ray-Based Spectroscopies. J. Phys. Chem. C.

[cit45] Rosenthal D., Ruta M., Schlögl R., Kiwi-Minsker L. (2010). Combined XPS and TPD Study of Oxygen-Functionalized Carbon Nanofibers Grown on Sintered Metal Fibers. Carbon.

[cit46] Stradella L., Argentero M. (1993). A Study of the Thermal Decomposition of Urea, of Related Compounds and Thiourea Using DSC and TG-EGA. Thermochim. Acta.

[cit47] Schaber P. M., Colson J., Higgins S., Thielen D., Anspach B., Brauer J. (2004). Thermal Decomposition (Pyrolysis) of Urea in an Open Reaction Vessel. Thermochim. Acta.

[cit48] Liu J., Zhang T., Wang Z., Dawson G., Chen W. (2011). Simple Pyrolysis of Urea into Graphitic Carbon Nitride with Recyclable Adsorption and Photocatalytic Activity. J. Mater. Chem..

[cit49] Ehrat F., Bhattacharyya S., Schneider J., Löf A., Wyrwich R., Rogach A. L., Stolarczyk J. K., Urban A. S., Feldmann J. (2017). Tracking the Source of Carbon Dot Photoluminescence: Aromatic Domains versus Molecular Fluorophores. Nano Lett.

[cit50] Barbooti M. M., Al-Sammerrai D. A. (1986). Thermal Decomposition of Citric Acid. Thermochim. Acta.

[cit51] Sun R., Wang Y., Ni Y., Kokot S. (2014). Graphene Quantum Dots and the Resonance Light Scattering Technique for Trace Analysis of Phenol in Different Water Samples. Talanta.

[cit52] Martindale B. C. M., Hutton G. A. M., Caputo C. A., Reisner E. (2015). Solar Hydrogen Production Using Carbon Quantum Dots and a Molecular Nickel Catalyst. J. Am. Chem. Soc..

[cit53] Biniak S., Szymański G., Siedlewski J., Świątkowski A. (1997). The Characterization of Activated Carbons with Oxygen and Nitrogen Surface Groups. Carbon.

[cit54] Darmstadt H., Roy C., Kaliaguine S. (1994). ESCA Characterization of Commercial Carbon Blacks and of Carbon Blacks from Vacuum Pyrolysis of Used Tires. Carbon.

[cit55] Figueiredo J. L., Pereira M. F. R. (2010). The Role of Surface Chemistry in Catalysis with Carbons. Catal. Today.

[cit56] Schuster M. E., Hävecker M., Arrigo R., Blume R., Knauer M., Ivleva N. P., Su D. S., Niessner R., Schlögl R. (2011). Surface Sensitive Study To Determine the Reactivity of Soot with the Focus on the European Emission Standards IV and VI. J. Phys. Chem. A.

[cit57] Halgren T. A. (1996). Merck Molecular Force Field. I. Basis, Form, Scope, Parameterization, and Performance of MMFF94. J. Comput. Chem..

[cit58] Molecular Operating Environment Version 2008.10, Chem. Comput. Gr., 2008

[cit59] Frisch M. J.; Trucks G. W., Schlegel H. B., Scuseria G. E., Robb M. A., Cheeseman J. R., Scalmani G., Barone V., Petersson G. A., Nakatsuji H., et al. Gaussian 16, Revision C.01, Gaussian, Inc., Wallingford CT, 2019

[cit60] Zhao Y., Truhlar D. G. (2006). Comparative DFT Study of van Der Waals Complexes: Rare-Gas Dimers, Alkaline-Earth Dimers, Zinc Dimer, and Zinc-Rare-Gas Dimers. J. Phys. Chem. A.

[cit61] Weigend F. (2006). Accurate Coulomb-Fitting Basis Sets for H to Rn. Phys. Chem. Chem. Phys..

[cit62] Grimme S., Antony J., Ehrlich S., Krieg H. (2010). A Consistent and Accurate Ab Initio Parametrization of Density Functional Dispersion Correction (DFT-D) for the 94 Elements H-Pu. J. Chem. Phys..

[cit63] Tomasi J., Mennucci B., Cammi R. (2005). Quantum Mechanical Continuum Solvation Models. Chem. Rev..

