# Supplementary material for: Carbon nanodots revised: the thermal citric acid/urea reaction
Source: Chem Sci. 2020 Jul 17;11(31):8256–66. doi: 10.1039/d0sc01605e (PMC8163031; doi:10.1039/d0sc01605e)
Supplement: SC-011-D0SC01605E-s001 [file SC-011-D0SC01605E-s001.pdf]

Electronic Supplementary Material (ESI) for Chemical Science.
This journal is © The Royal Society of Chemistry 2020

Supporting Information to

Volker Strauss1*, Huize Wang1, Simon Delacroix1, Marc Ledendecker2, Pablo Wessig3

Synthesis 24
Nuclear Magnetic Resonance Spectroscopy of the Raw Products 24
UV-vis and Fluorescence Spectroscopy of the Raw Products 25
Nuclear Magnetic Resonance Spectroscopy of the Educts and References 26
Separation of the Raw Products 27
Extinction coefficients of CUg 27
Time-correlated Single-Photon Counting of CUg and CUb 28
Fluorescence Quantum Yields of CUg 28
Photostability of CUg 29
ESI - mass spectrometry of CUg and CUb 29
2D-Correlation NMR of CUg 30
DFT calculations of CUg (HPPT) 31
2D-Correlation NMR of CUb 32
Nuclear Magnetic Resonance Spectroscopy of Biuret 33
Biuret test with CUb 33
Thermal Mass analysis of CUg and CUb 34
Thermogravimetric Analysis of the Raw Mixture 34
X-ray Photoelectron Spectroscopy of CUb and CUg 35
Nuclear Magnetic Resonance Spectroscopy during Washing of CUp 35
Optical Spectroscopy of dispersions of CUp in H_2O. 36
Nuclear Magnetic Resonance Spectroscopy of a Non-Separated Mixture 36

Synthesis

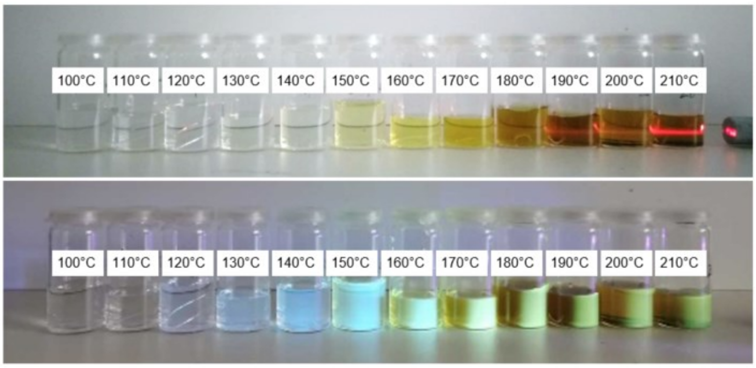

Figure S1. *Photograph of aqueous sample solutions of the crude products (conc: 0.2 mg/mL) synthesized at temperatures between 100 – 220°C under illumination with a red laser (top) and under illumination with a UV-lamp (365 nm) (bottom).*

Nuclear Magnetic Resonance Spectroscopy of the Raw Products

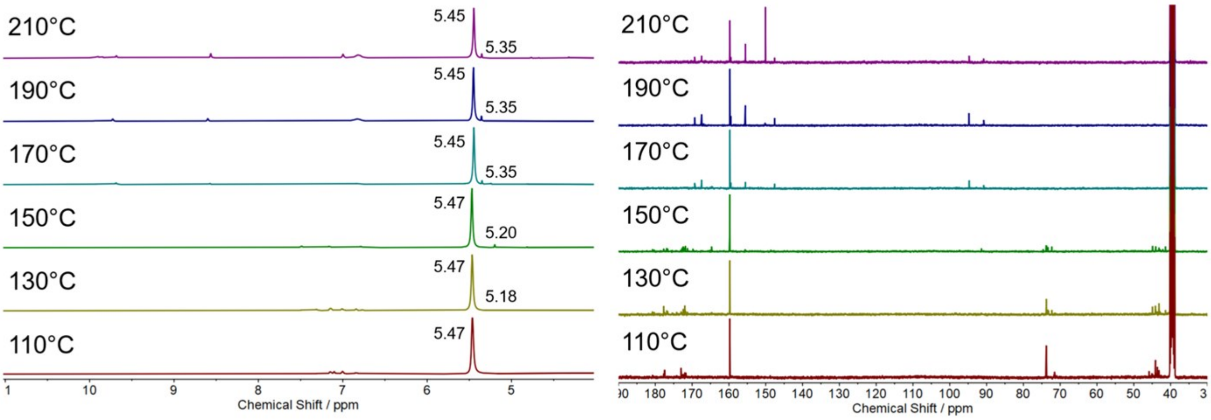

Figure S2. *^{1}H-NMR (left) and ^{13}C-NMR (right) spectra of selected raw products of the CA/U reaction at different temperatures between 110-210°C.*

UV-vis and Fluorescence Spectroscopy of the Raw Products

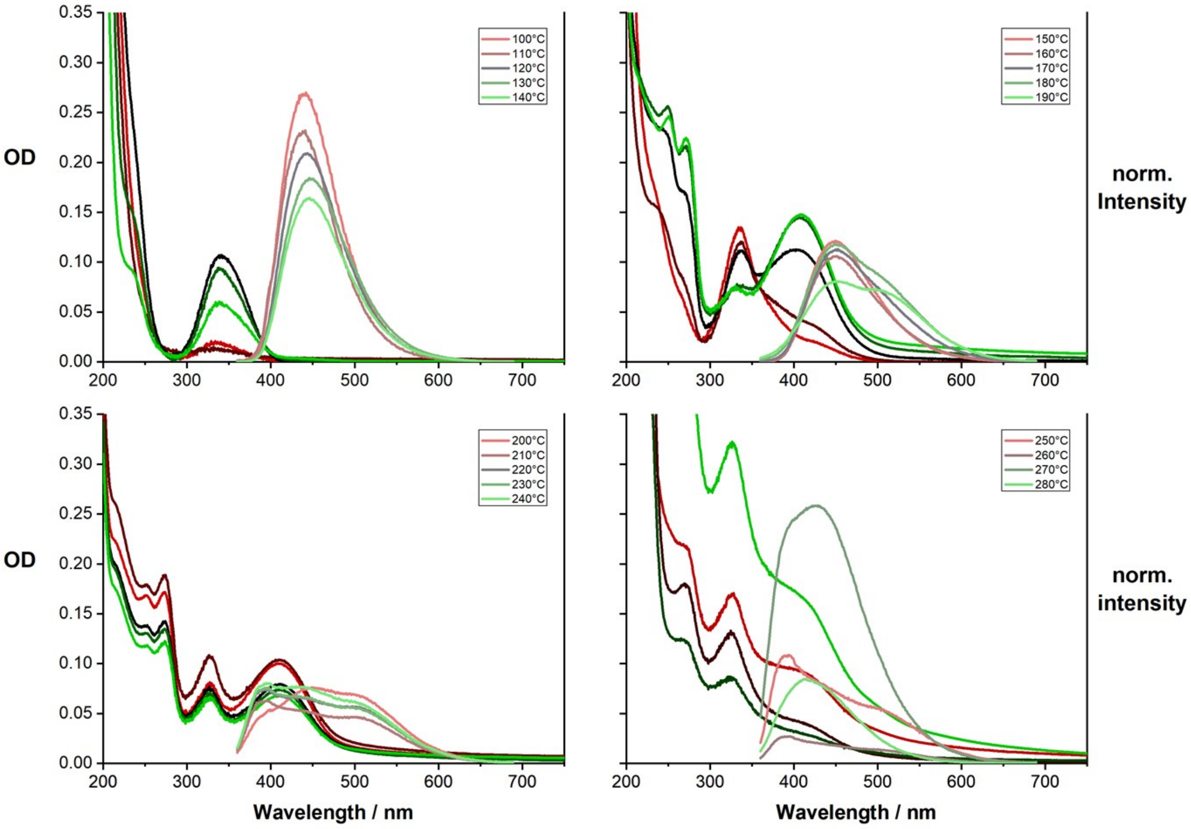

Figure S3. *Combined electronic absorption (red to green) and excitation normalized (λ_{ex} = 350 nm) emission (pale red to pale green) spectra of crude CA/U products (0.013 mg/mL) synthesized at different temperatures between 100 – 290°C; a) 100 – 140°C; b) 150 – 190°C; a) 200 – 240°C; a) 250 – 290°C.*

Nuclear Magnetic Resonance Spectroscopy of the Educts and References

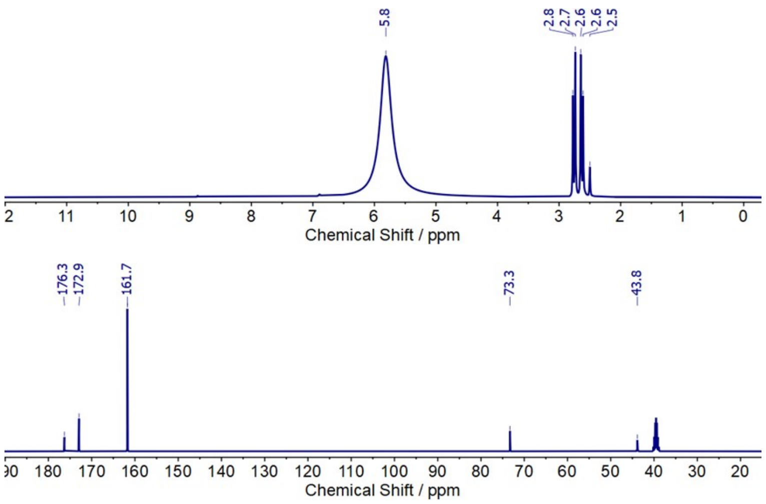

Figure S4. *Top: ^{1}H-NMR (top) and ^{13}C-NMR (bottom) spectra of* ***citric acid*** *and* ***urea*** *in DMSO-d6.*

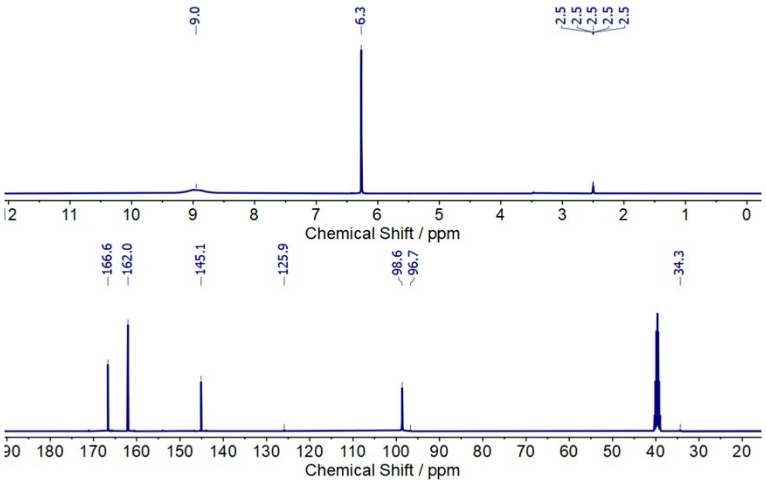

Figure S5. *Top: ^{1}H-NMR (top) and ^{13}C-NMR (bottom) spectra of* ***citrazinic acid*** *(commercial) in DMSO-d6.*

Separation of the Raw Products

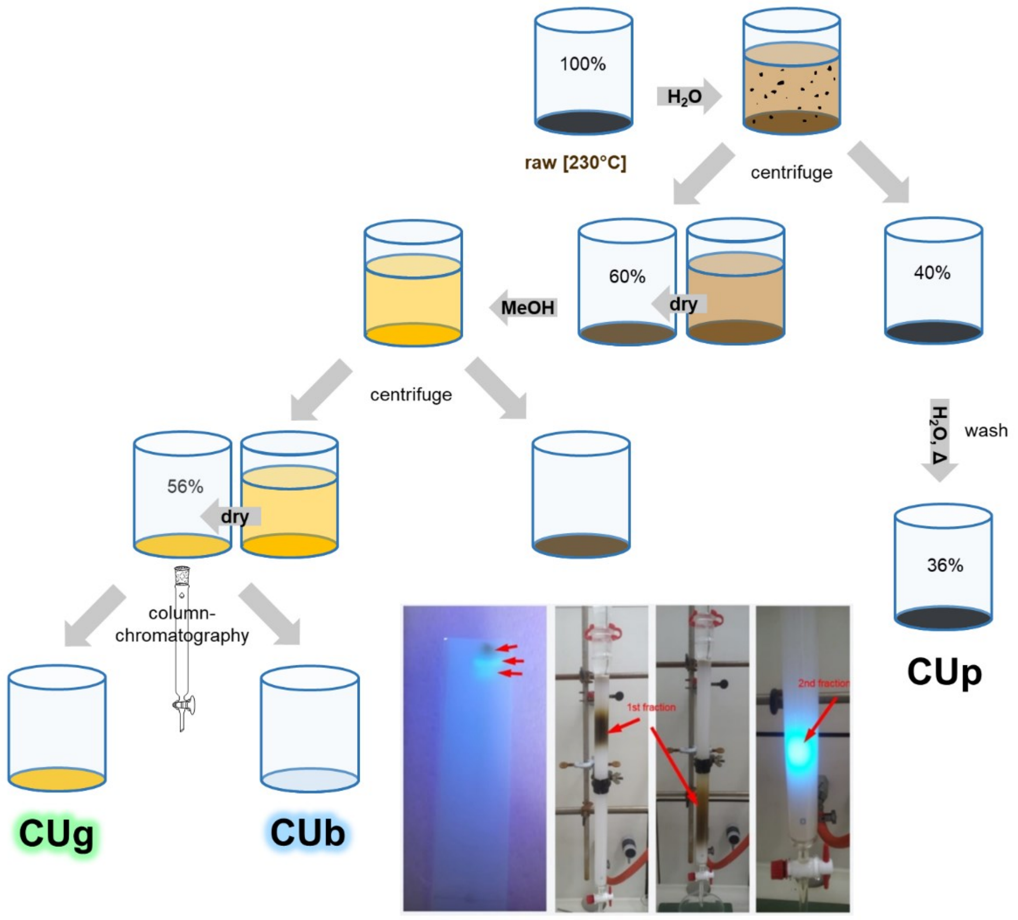

Figure S6. *Illustration of the separation process of the raw products obtained from a thermal CA/U reaction.*

Extinction coefficients of CUg

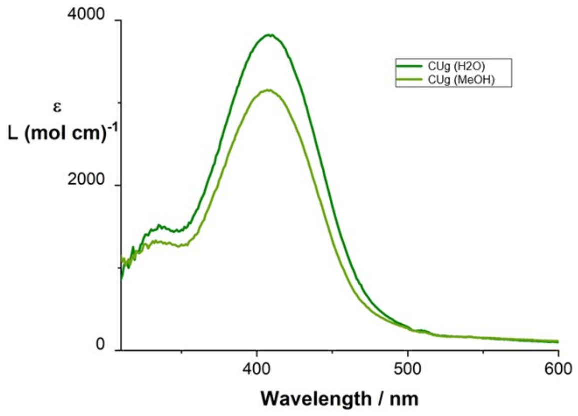

Figure S7. *Molar extinction coefficients of **CUg** in H_2O and MeOH.*

Time-correlated Single-Photon Counting of CUg and CUb

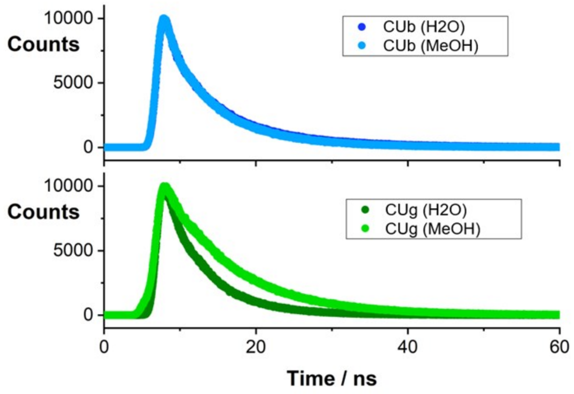

Figure S8. *Fluorescence lifetime decay profiles obtained by TCSPC of **CUb** and **CUg** in H_2O and MeOH at room temperature obtained upon excitation at 370 or 450 nm, respectively .*

Table S1. *Fluorescence lifetimes of **CUb** and **CUg** in MeOH and H_2O.*

		τ_1 ns	A_1 %	τ_2 ns	A_2 %
**CUb**	MeOH	3.1	28	7.6	72
	H_2O	4.1	45	9.5	55
**CUg**	MeOH	9.0	100		
	H_2O	5.3	100		

Fluorescence Quantum Yields of CUg

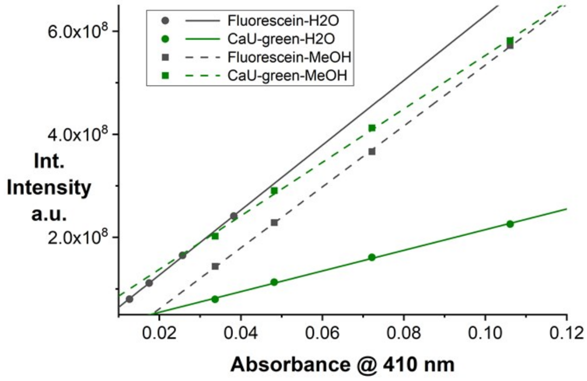

Figure S9. *Determination of the fluorescence quantum yields of **CUg** in H_2O and MeOH using Na-fluorescein as a standard.*

__Table S2.__ Fluorescence quantum yields of __CUg__ determined by the gradient method using Na-fluorescein as a standard and in MeOH and H_2O.

	solvent	slope	φ_{Fl} (%)
Na-Fluorescein	H_2O	6.30E+09	**85**[64]
	MeOH	5.90E+09	**79**
**CUg**	H_2O	2.00E+09	**27**
	MeOH	5.20E+09	**70**

Photostability of CUg

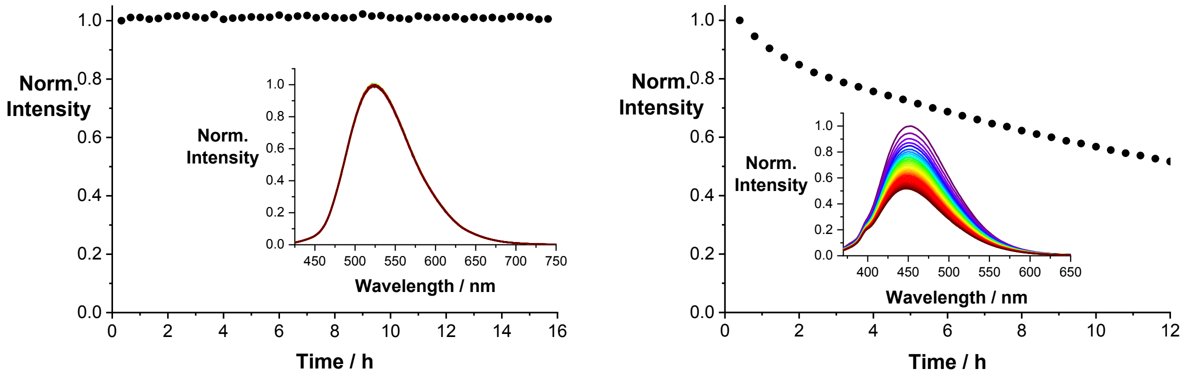

__Figure S10.__ Left: photo-stability measurement of a 8.3×10^{-5} M solution of __CUg__ in H_2O. The sample was constantly illuminated for 16h. The inset shows the fluorescence spectra in time intervals of 20 min; Right: Photo-stability measurement of __CUb__ in H_2O. The inset shows the fluorescence spectra in time intervals of 24 min from purple to red.

ESI - mass spectrometry of CUg and CUb

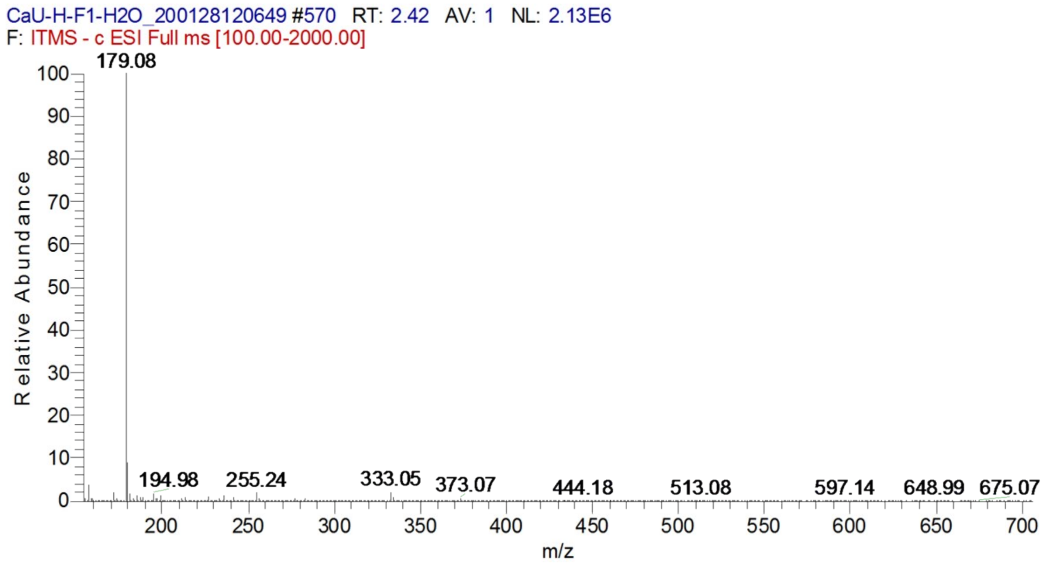

__Figure S11.__ Liquid chromatography-electrospray ionization - mass spectrum of __CUg__ obtained in negative ion mode.

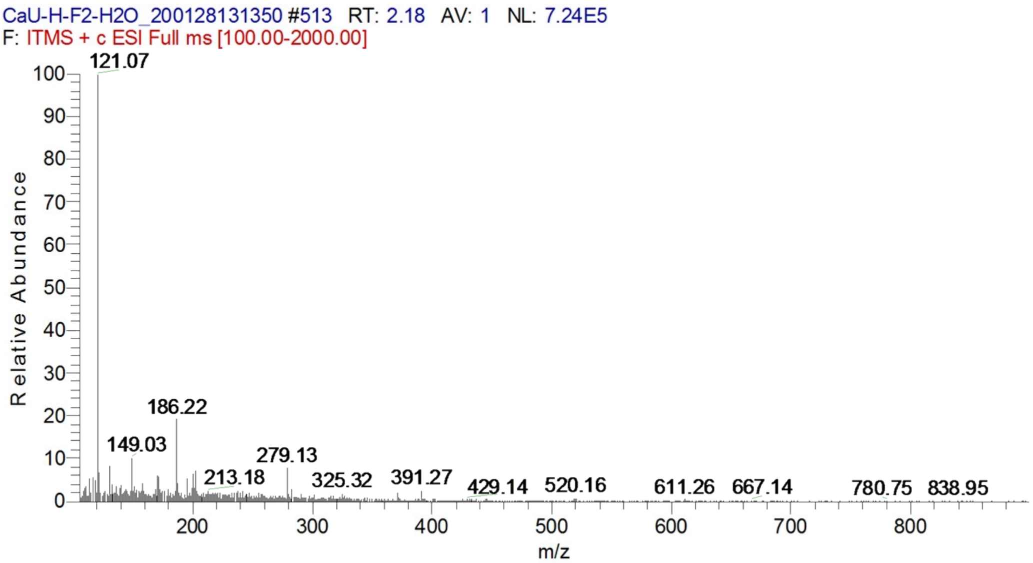

Figure S12. Liquid chromatography – electrospray ionization - mass spectrum of **CUb** obtained in positive ion mode.

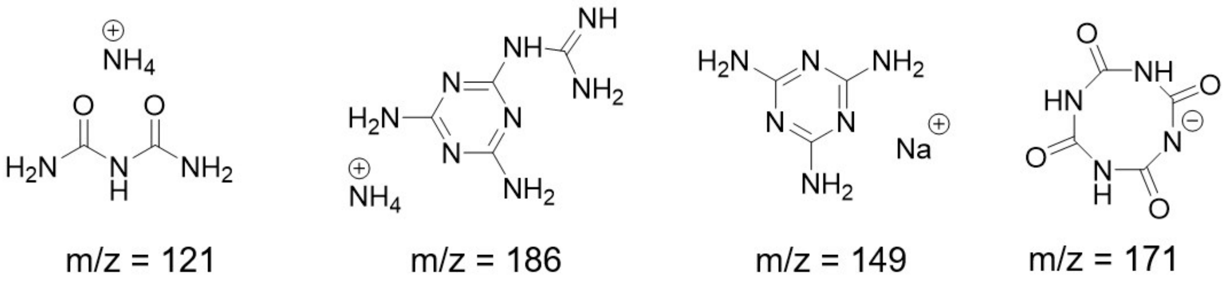

Figure S13. Potential compounds detected in the ESI-mass spectra of CUb.

2D-Correlation NMR of CUg

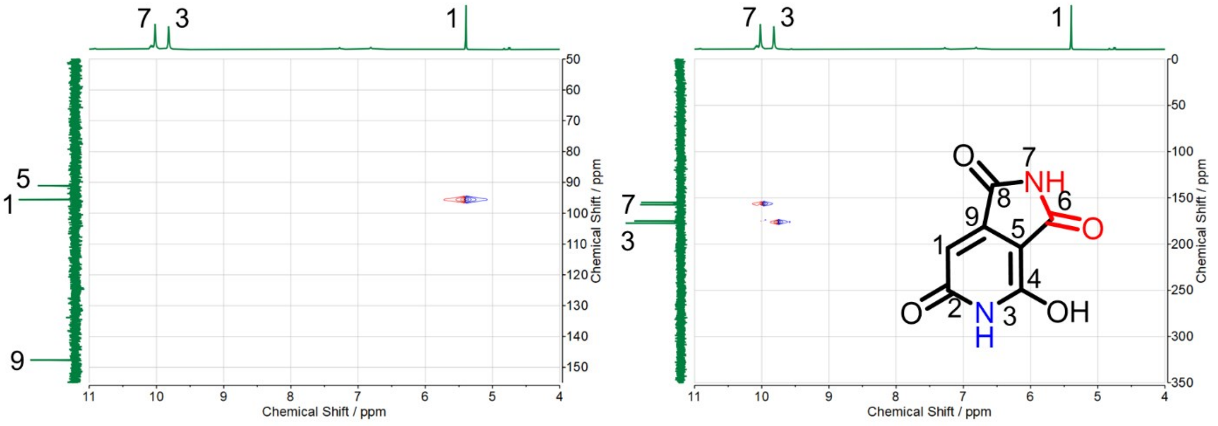

Figure S14. HSQC-C (left) and HSQC-N (right) of **CUg** in DMSO-d6 showing the direct coupling of ^{1}H to ^{13}C or ^{15}N, respectively.

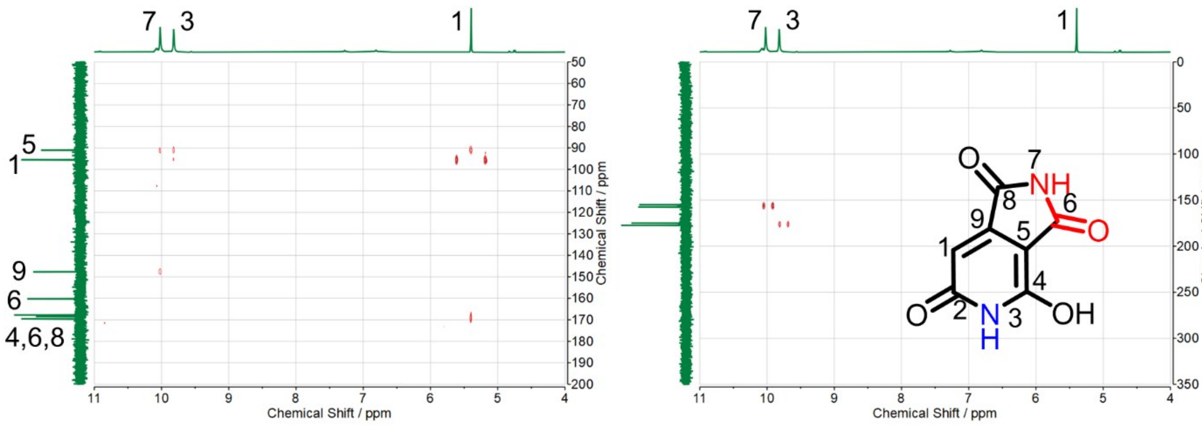

Figure S15. *HMBC-C (left) and HMBC-N (right)* ***CUg*** *enriched with* ^{15}*N in DMSO-d6 showing the three-bond coupling of* ^{1}*H to* ^{13}*C or* ^{15}*N, respectively.*

DFT calculations of CUg (HPPT)

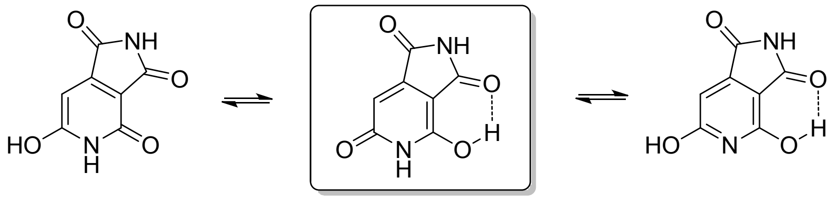

A B C

Figure S16*. Molecular structures of the tautomeric forms of HPPT.*

Table S3. *DFT calculated tautomeric forms of HPPT with relative energy values in arbitrary units using M06-2X-D3/def2-TZVP, SCRF: PCM.*

	Geometry (vacuum)	Geometry (MeOH)	Geometry (H_2O)	E_{REL} (vac.)	E_{REL} (MeOH)	E_{REL} (H_2O)
**A**	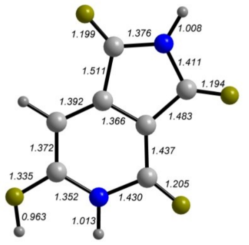	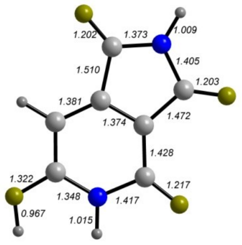	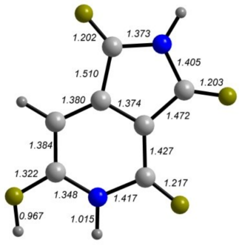	+10.9	+ 4.4	+ 4.2

**B**	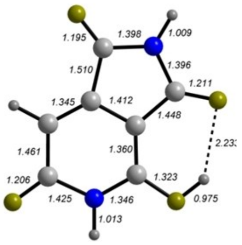	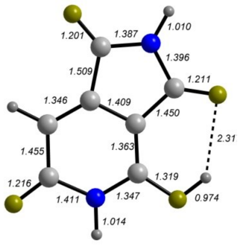	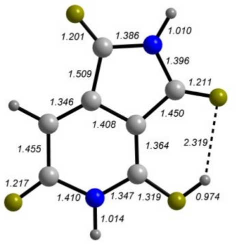	**± 0**	**± 0**	**± 0**
**C**	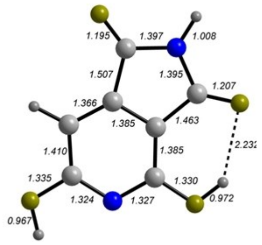	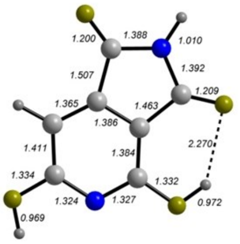	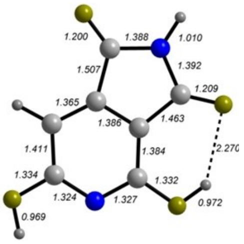	-0.4	+ 1.6	+ 1.6

2D-Correlation NMR of CUb

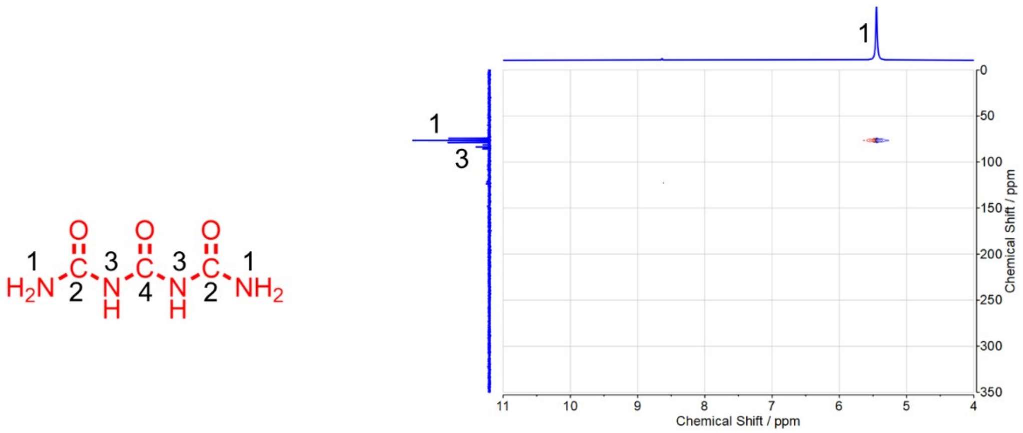

Figure S17. *HSQC-N of **CUb** enriched with ^{15}N in DMSO-d6 showing the direct coupling of ^{1}H to ^{13}C or ^{15}N, respectively.*

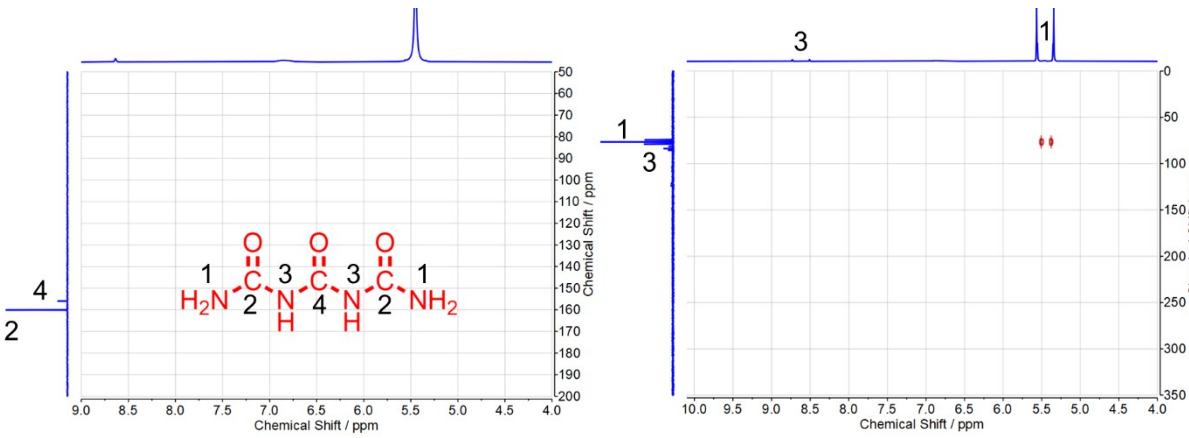

Figure S18. *HMBC-C (left) and HMBC-N (right) of* ***CUb*** *enriched with* ^{15}N *in DMSO-d6 showing the three-bond coupling of* ^{1}H *to* ^{13}C *or* ^{15}N*, respectively.*

Nuclear Magnetic Resonance Spectroscopy of Biuret

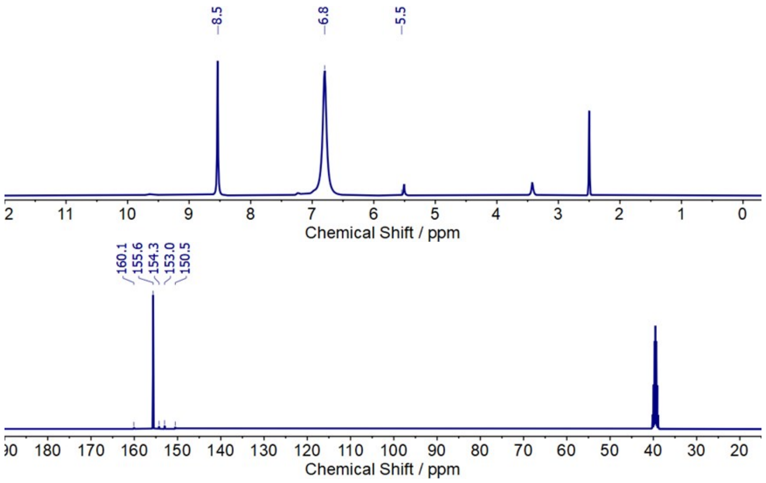

Figure S19. *Top:* ^{1}H*-NMR (top) and* ^{13}C*-NMR (bottom) spectra of* ***biuret*** *(commercial) in DMSO-d6.*

Biuret test with CUb

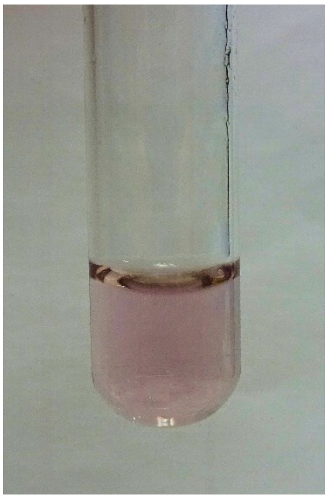

Figure S20. *Photograph of a basic solution of* ***CUb*** *upon addition of a* $CuSO_4$ *solution.*

Thermal Mass analysis of CUg and CUb

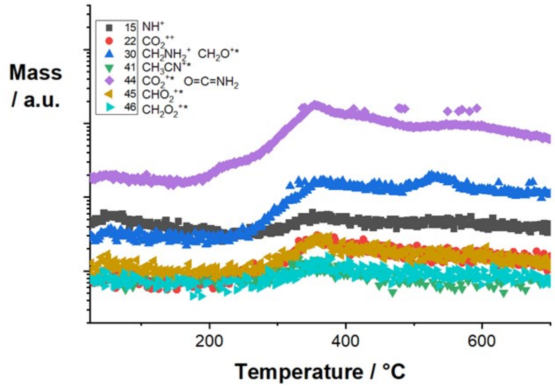

Figure S21. *Temperature dependent mass spectrometric analysis of* ***CUg***.

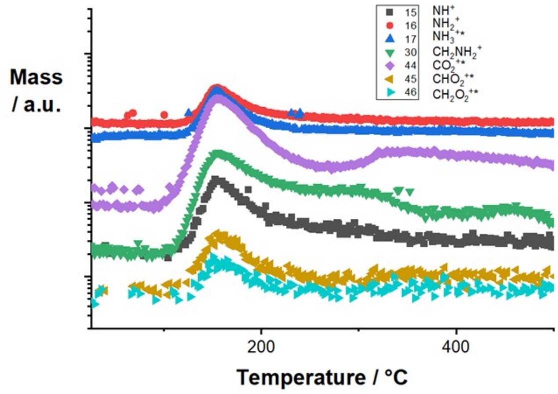

Figure S22. *Temperature dependent mass spectrometric analysis of* ***CUb***.

Thermogravimetric Analysis of the Raw Mixture

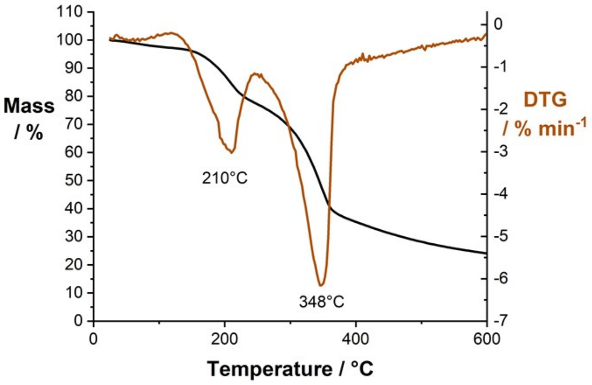

Figure S23. *Thermogravimetric analysis of the supernatant of the raw product before column chromatography containing* ***CUg*** *and* ***CUb.***

X-ray Photoelectron Spectroscopy of CUb and CUg

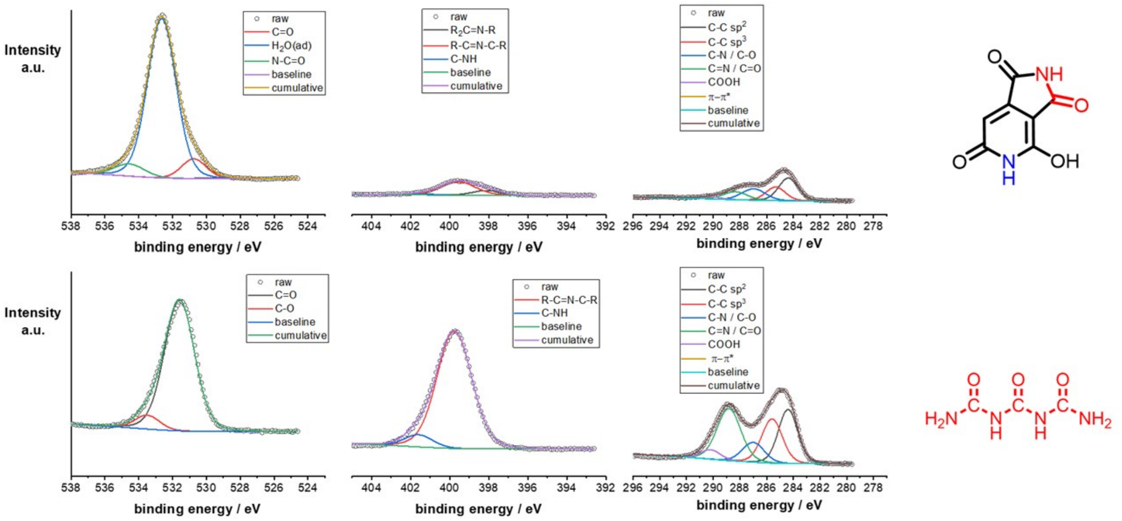

Figure S24. *X-ray photoelectron spectra of* ***CUg*** *(top) and* ***CUb*** *(bottom) with emphasis on the* C_{1s}*,* N_{1s}*, and* O_{1s} *regions.*

Nuclear Magnetic Resonance Spectroscopy during Washing of CUp

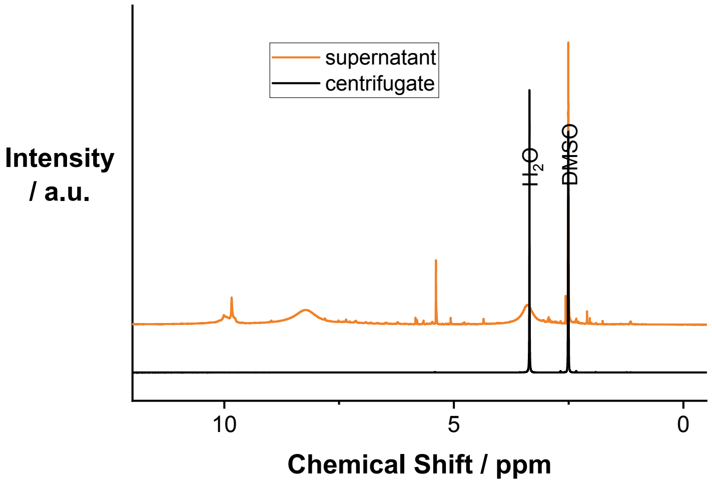

Figure S25. *^{1}H-NMR spectra of the supernatant and the redispersed centrifugate after washing* ***CUp*** *in* H_2O *at 95°C in DMSO-d6.*

Optical Spectroscopy of dispersions of CUp in H_2O.

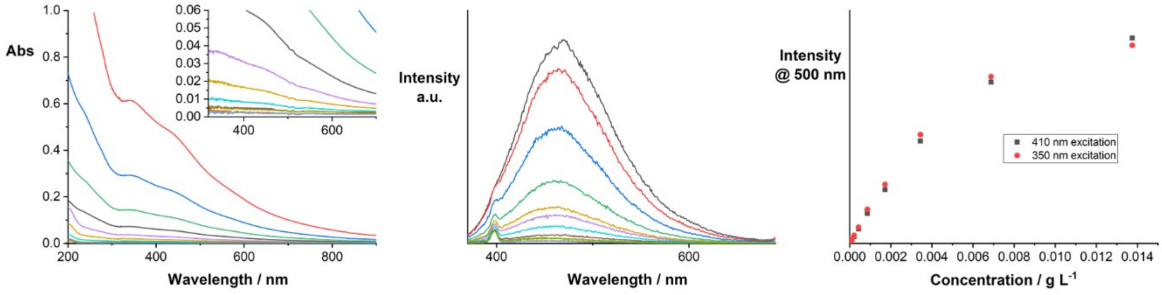

Figure S26. *Absorption (left) and fluorescence (center) spectra of **CUp** in H_2O at room temperature at different concentrations. Right: Fluorescence intensity versus concentration of **CUp** in H_2O.*

Nuclear Magnetic Resonance Spectroscopy of a Non-Separated Mixture

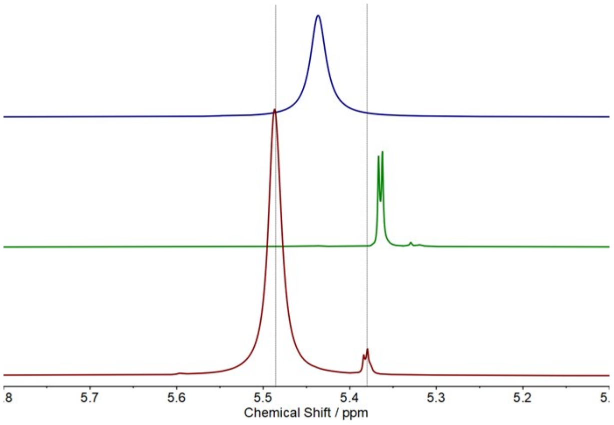

Figure S27. *^{1}H-NMR spectra of **CUb** (top), **CUg** (center) and the non-separated mixture of **CUb** and **CUg** (bottom) in DMSO-d6.*

References (continued from paper)

64. X.-F. Zhang, J. Zhang and L. Liu, Fluorescence Properties of Twenty Fluorescein Derivatives: Lifetime, Quantum Yield, Absorption and Emission Spectra, *J. Fluoresc.*, 2014, **24** (3), 819–826, 10.1007/s10895-014-1356-5
